# Risk and Outcome of Breakthrough COVID-19 Infections in Vaccinated Patients With Cancer: Real-World Evidence From the National COVID Cohort Collaborative

**DOI:** 10.1200/JCO.21.02419

**Published:** 2022-03-14

**Authors:** Qianqian Song, Benjamin Bates, Yu Raymond Shao, Fang-Chi Hsu, Feifan Liu, Vithal Madhira, Amit Kumar Mitra, Timothy Bergquist, Ramakanth Kavuluru, Xiaochun Li, Noha Sharafeldin, Jing Su, Umit Topaloglu

**Affiliations:** ^1^ Wake Forest School of Medicine, Winston-Salem, NC; ^2^ Rutgers University, New Brunswick, NJ; ^3^ Duke University Medical Center, Durham, NC; ^4^ University of Massachusetts Chan Medical School, Boston, MA; ^5^ Palila Software LLC, Reno, NV; ^6^ Harrison School of Pharmacy, Auburn University, Auburn, AL; ^7^ Sage Bionetworks, Seattle, WA; ^8^ University of Kentucky, Lexington, KT; ^9^ Department of Biostatistics and Health Data Science, Indiana University School of Medicine, Indianapolis, IN; ^10^ School of Medicine, University of Alabama at Birmingham, Birmingham, AL

## Abstract

**PURPOSE:**

To provide real-world evidence on risks and outcomes of breakthrough COVID-19 infections in vaccinated patients with cancer using the largest national cohort of COVID-19 cases and controls.

**METHODS:**

We used the National COVID Cohort Collaborative (N3C) to identify breakthrough infections between December 1, 2020, and May 31, 2021. We included patients partially or fully vaccinated with mRNA COVID-19 vaccines with no prior SARS-CoV-2 infection record. Risks for breakthrough infection and severe outcomes were analyzed using logistic regression.

**RESULTS:**

A total of 6,860 breakthrough cases were identified within the N3C-vaccinated population, among whom 1,460 (21.3%) were patients with cancer. Solid tumors and hematologic malignancies had significantly higher risks for breakthrough infection (odds ratios [ORs] = 1.12, 95% CI, 1.01 to 1.23 and 4.64, 95% CI, 3.98 to 5.38) and severe outcomes (ORs = 1.33, 95% CI, 1.09 to 1.62 and 1.45, 95% CI, 1.08 to 1.95) compared with noncancer patients, adjusting for age, sex, race/ethnicity, smoking status, vaccine type, and vaccination date. Compared with solid tumors, hematologic malignancies were at increased risk for breakthrough infections (adjusted OR ranged from 2.07 for lymphoma to 7.25 for lymphoid leukemia). Breakthrough risk was reduced after the second vaccine dose for all cancers (OR = 0.04; 95% CI, 0.04 to 0.05), and for Moderna's mRNA-1273 compared with Pfizer's BNT162b2 vaccine (OR = 0.66; 95% CI, 0.62 to 0.70), particularly in patients with multiple myeloma (OR = 0.35; 95% CI, 0.15 to 0.72). Medications with major immunosuppressive effects and bone marrow transplantation were strongly associated with breakthrough risk among the vaccinated population.

**CONCLUSION:**

Real-world evidence shows that patients with cancer, especially hematologic malignancies, are at higher risk for developing breakthrough infections and severe outcomes. Patients with vaccination were at markedly decreased risk for breakthrough infections. Further work is needed to assess boosters and new SARS-CoV-2 variants.

## INTRODUCTION

There is an urgent demand for real-world evidence (RWE) on the efficacy of COVID-19 vaccines in patients with cancers. This vulnerable population is disproportionately and heterogeneously affected by the COVID-19 pandemic. Significantly higher infection risk and higher overall mortality in specific cancers including hematologic and lung cancers have been reported by our team^1^ and others.^2-4^ COVID-19 vaccines have demonstrated high efficacy in preventing infection and severe outcomes according to recent clinical trials, observational studies, and RWE in the general population.^5-8^ Limited evidence, however, exists on the effectiveness of COVID-19 vaccines in patients with cancers.^9^ Immune competence varies across cancer types and treatments, which may result in disparate responses to COVID-19 vaccines. In particular, the immunosuppressive states associated with certain types of cancers (eg, hematologic malignancies^10^) and cancer treatments (eg, anti–B-cell therapies and proteasome inhibitor therapies^11^) can impair acquired immune responses to vaccines. Existing evidence showed that antibody titers in the patients with cancer of immunosuppressive states are significantly lower^12^; nevertheless, COVID-19 vaccines have demonstrated a strong T-cell response and may provide protective T-cell immunity regardless of antibody titers.^13^ However, cancer-specific clinical trials such as the VOICE (vaccination against COVID in cancer) study have just been launched in recent months.^14^ The current public health policies of providing COVID-19 vaccines, including booster doses, to patients with cancers are mainly based on the hypothesis that the benefits of vaccination outweigh their risks.^15^ To our knowledge, a large-scale, comprehensive investigation on the effectiveness and heterogeneity of COVID-19 vaccines in patients with cancers still does not exist.

**CONTEXT**

**Key Objective**
Immune competence varies across cancer types and treatments, which may result in disparate responses to COVID-19 vaccines. Our study aims to generate clinically actionable knowledge about the vaccine effectiveness for the heterogeneous and vulnerable cancer population, using a nationally representative cohort of patients made available through the National COVID Cohort Collaborative (N3C) consortium.
**Knowledge Generated**
In the N3C-vaccinated population of 6,860 breakthrough cases, hematologic malignancies and solid tumors demonstrated significantly higher risks for breakthrough infection (odds ratios = 4.64 and 1.12) and severe outcomes (odds ratios = 1.45 and 1.33) after adjusting for age, sex, race and ethnicity, smoking status, vaccine type, and vaccination date. Patients with recent cancer treatment showed higher risks of breakthrough infections.
**Relevance** **(*J.W. Friedberg*)**
This work provides one of the largest national-level real-world evidence on risks of COVID-19 breakthrough infections and subsequent outcomes, confirming prior risk groups. Vaccinations against COVID-19 remain protective, and future studies will define the impact of boosters and monoclonal antibody prophylaxis in the most vulnerable populations as defined in this study.*
  *Relevance section written by *JCO* Editor-In-Chief Jonathan W. Friedberg, MD.

Current evidence suggests that, although mRNA-1273 (Moderna) and BNT162b2 (Pfizer–BioNTech) vaccines show more than 90% efficacy in preventing COVID-19^16,17^ after the second dose, Moderna's vaccine is shown to generate more than double the antibodies than Pfizer's vaccine.^18^ Another study shows that breakthrough infections are less likely to occur among those vaccinated with Moderna compared with Pfizer,^19^ and the rate of hospitalization was lower among the Moderna-vaccinated cohort versus the Pfizer cohort. This indicates that Moderna's vaccine may provide better protection for immunocompromised people including patients with cancer. However, corresponding RWE for the population with cancers is still missing.

Our study aims to address these knowledge gaps using a large, nationally representative cohort of patients made available through the National COVID Cohort Collaborative (N3C) consortium.^20^ The N3C enclave houses the largest harmonized and integrated clinical cohort registry of COVID-19–tested patients in the United States and includes electronic health record data of approximately 7.9 million patients with one (or more) clinical encounters after January 1, 2020 (inpatient or outpatient), from more than 65 US medical centers. Our work provides one of the first and the largest national-level RWE on risk for COVID-19 breakthrough infections in patients with cancers, measures the effectiveness of mRNA vaccines in preventing breakthrough infections, and outlines the outcomes of breakthrough cases.

## METHODS

### Study Cohort

We defined our breakthrough analytic cohort as patients who have (1) received at least one dose of an mRNA vaccine between December 01, 2020, and May 31, 2021 (BNT162b2 by Pfizer-BioNTech or mRNA-1273 by Moderna); (2) never been diagnosed with COVID-19 before vaccination; and (3) developed COVID-19 at least 14 days from the first mRNA vaccine dose. Those who had received two doses of vaccines before COVID-19 infection or remained uninfected by the cutoff date were considered as fully vaccinated, whereas those who only received one dose were defined as partially vaccinated. The COVID-19 infection status (positive or negative) was identified according to diagnosis records, reverse transcription polymerase chain reaction testing results, and COVID-19 antibody testing results (Data Supplement, online only) as previously described.^1^

### Indicator Variables

We included data on age at the time of the first vaccine dose, sex, race and ethnicity, and smoking status (Data Supplement). We used available data to calculate indicator variables on the Charlson Comorbidity Index (CCI)^21^ adjusted for the cancer diagnosis. Comorbidity categories were defined by mapping the International Classification of Diseases ICD-10-Clinical Modification codes used in the definition by Quan et al to each code's Observational Medical Outcomes Partnership standard equivalent Systematized Nomenclature of Medicine (SNOMED) code.^21-23^ The geographic variance and potential impacts were also examined.

### Primary Cancer Diagnosis

Cancer diagnosis algorithm^1^ is provided in the Data Supplement. Patients with cancers within the N3C registry were identified by the Malignant Neoplastic Disease standard concept (SNOMED Code: 363346000) using the Observational Health Data Sciences and Informatics ATLAS tool.

### Cancer Therapies

Exposure to systemic, nontopical cancer therapies was assessed for each patient in our cohort using a string search for each cancer therapy in the drug concept name and manually reviewed for correctness. Cancer therapies belonging to 15 major drug classes in the National Cancer Institute Division of Cancer Control and Population Sciences (Data Supplement)^24,25^ were identified. Bone marrow transplantation before vaccination was also identified according to N3C Concept Set 5960049. This concept set included the vocabulary descendants of the SNOMED code 42537745 (bone marrow transplant present) and the SNOMED code 23719005 (transplantation of bone marrow). Medications with major immunosuppressive effects were identified through cross-referencing Physicians' Cancer Chemotherapy Drug Manual 2021 (ISBN 1284230139) and The Washington Manual Hematology and Oncology Subspecialty Consult (ISBN 1496328086).

### Outcomes

The primary outcome of interest was a COVID-19 infection after 14 days of receiving the first or the second dose of an mRNA vaccine. Severe COVID-19 outcomes were defined as death (including discharge to hospice), hospitalization, or use of mechanical ventilation or extracorporeal membrane oxygenation.

### Statistical Analysis and Visualization

Descriptive analyses were shown with counts and percentages for categorical variables and with medians and the corresponding interquartile ranges (IQRs) for numeric variables. The vaccinated patients were identified following CDC guidelines.^26^ Risks for breakthrough infection and severe outcomes were evaluated using multivariable logistic regression models. The models were adjusted for age group at vaccination date, sex, race and ethnicity, smoking status, vaccination type (fully or partially vaccinated), vaccine types (BNT162b2 by Pfizer-BioNTech or mRNA-1273 by Moderna), primary cancer types, recent cancer treatment, and adjusted CCI variables (for outcome analysis). Adjusted odds ratios (ORs) with an adjusted 95% CI were estimated for these potential risk factors. Two-tailed *P* values were calculated using the Wald test. For cancer drugs analysis, the *P* values and 95%CIs were adjusted for multiple testing using false discovery rates.^27^ Explained variations and goodness of fit of models were comprehensively evaluated, and the results are provided in the Data Supplement.

Per N3C policy, exact counts that are 20 or less are not reported to protect the privacy of individuals. All analyses are performed in the N3C Data Enclave on the Palantir platform.

### The Role of the Institutional Review Board

Data received by the National Center for Advancing Translational Sciences for the N3C Data Enclave are covered under a National Institutes of Health Institutional Review Board (IRB)–approved protocol with waiver of consent for Electronic Health Record–derived COVID-19–related research. All the authors who performed analyses and had access to N3C data in the Enclave obtained individual institutional review board approvals from their respective institutions for this project and were also approved to use a limited data set by the N3C Data Use Request Committee.

## RESULTS

### Cohort Collection

**FIG 1.**
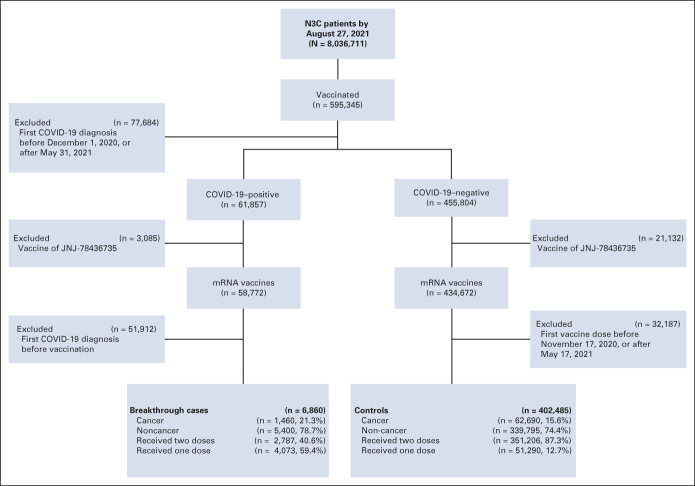
CONSORT diagram. The whole N3C-vaccinated population was screened according to the N3C data released on August 27, 2021. The exclusion criteria were based on scientific needs (excluding patients who had COVID-19 infections before vaccinations) and data availability (excluding JNJ-78436735), completeness (excluding COVID-19 infections after May 31, 2021), and quality. N3C, National COVID Cohort Collaborative.

As of August 27, 2021, our N3C cohort (data release version 42) consists of 58,772 COVID-19–positive cases with vaccination records (median age of 50 years, 58.2% female, 46.2% non-Hispanic White, and 11.0% with four or more comorbidities). Among this vaccination cohort, there were 6,860 breakthrough cases, with 2,787 fully vaccinated and 4,073 partially vaccinated cases (Fig 1). Meanwhile, there were 402,485 in the control group without COVID-19 infection records, including 351,206 fully vaccinated and 51,290 partially vaccinated cases. We noticed a significantly lesser proportion of fully vaccinated patients in the breakthrough infection group (40.6%) than in the control group (87.3%), with an OR of 0.10 (Fisher's exact test: *P* value ≤ .0001). Of note, the 6,860 breakthrough cases included 1,460 patients with cancer, with 867 fully and 593 partially vaccinated. Cancer cases demonstrated a significantly greater proportion of fully vaccinated patients (59.4%) with breakthrough infection than noncancer cases (35.6%), showing an OR of 2.65 (*P* value of Fisher's exact test: < .0001).

### Demographic and Clinical Characteristics

Among breakthrough cases (Table 1), the fully vaccinated subgroup had a higher median age of 63 years and higher proportions of severe outcomes (37.7%), current or former smokers (40.3%), and four or more comorbidities (22.2%) than the partially vaccinated subgroup. The cancer breakthrough cases demonstrated similar trends. Skin cancer and leukemia were top solid and hematologic cancer types. Consistently, among all vaccinated while not infected cases (Table 1), there were higher proportions of severe outcomes (32.6%), current or former smokers (31.5%), and more cases of four or more comorbidities (16.3%) in the fully vaccinated subgroup.

**TABLE 1.**
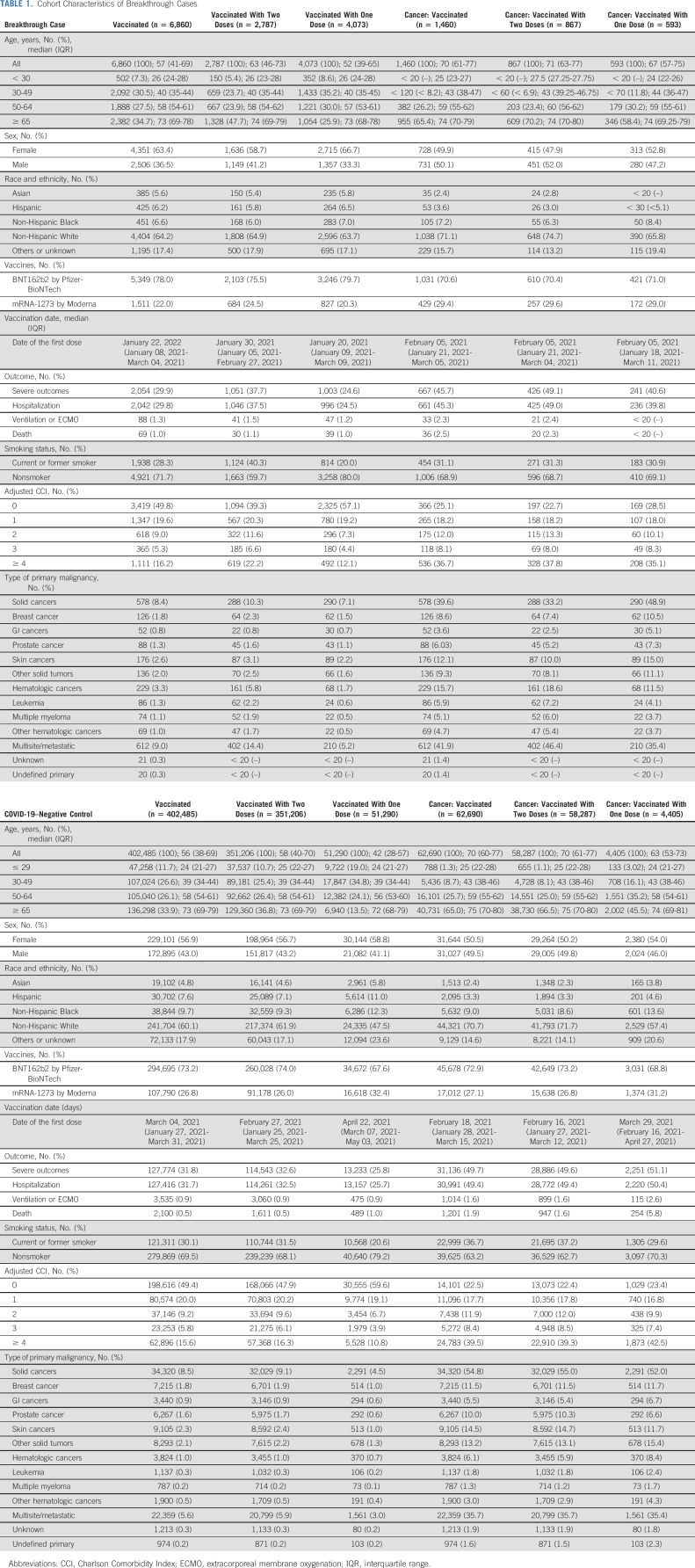
Cohort Characteristics of Breakthrough Cases

### Vaccination Effectiveness in Protecting Patients With Cancer from COVID-19 Infection

**FIG A1.**
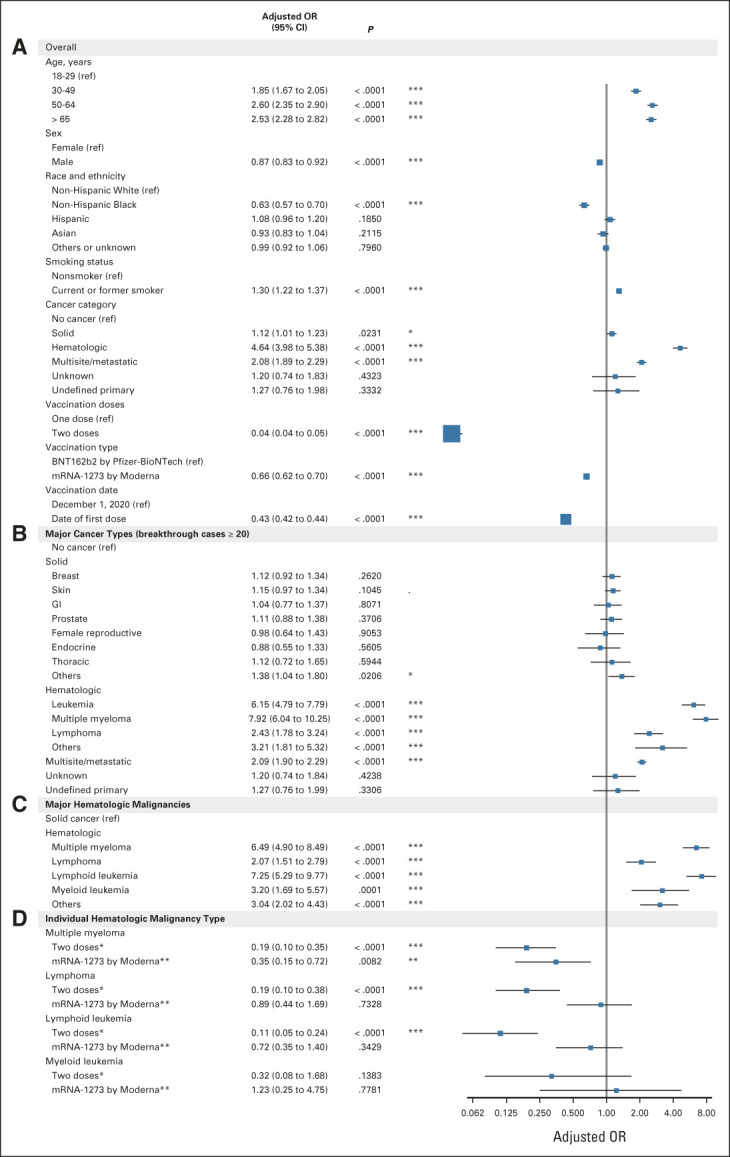
The mRNA vaccination effectiveness in protecting patients with cancer from COVID-19 infection. The forest plots of logistic linear regression analyses are shown for (A) all features in the overall breakthrough cases and negative controls; (B) major cancer types in the overall breakthrough cases and negative controls; (C) major hematologic malignancies (breakthrough cases ≥ 20) in patients with cancers; and (D) fully vaccinated versus partially vaccinated cases and mRNA-1273 versus BNT162b2 vaccines in four individual patient groups with multiple myeloma, lymphoma, lymphoid leukemia, and myeloid leukemia. Age, sex, race and ethnicity, smoking status, vaccination doses, vaccination types, and vaccination date were included in all logistic regression models. Individual logistic regression analyses were performed for each hematologic malignancy type, and the results are summarized in (D). *P < .05, **P < .01, ***P < .001. OR, odds ratio; ref, reference.

As shown in Appendix Figure A1A (online only) and the Data Supplement, age over 65 years (adjusted OR: 2.53; 95% CI, 2.28 to 2.82), current or former smokers (adjusted OR: 1.3; 95% CI, 1.22 to 1.37), solid cancers (adjusted OR: 1.12; 95% CI, 1.01 to 1.23), and hematologic cancers (adjusted OR: 4.64; 95% CI, 3.98 to 5.38) were associated with increased risk of breakthrough infections. Male (adjusted OR: 0.87; 95% CI, 0.83 to 0.92) and non-Hispanic Black (adjusted OR = 0.63; 95% CI, 0.57 to 0.7) were at lower risk. Full vaccination (adjusted OR: 0.04; 95% CI, 0.04 to 0.05), Moderna's vaccine (adjusted OR: 0.66; 95% CI, 0.62 to 0.7), and vaccination calendar date (adjusted OR: 0.43; 95% CI, 0.42 to 0.44) showed lower risk of breakthrough infections.

Individual multivariable analysis results for specific cancer types and potential risk factors are shown in Appendix Figure A1B and the Data Supplement. Compared with noncancer cases, patients with hematologic cancers including leukemia (adjusted OR: 6.15; 95% CI, 4.79 to 7.79), multiple myeloma (adjusted OR: 7.92; 95% CI, 6.04 to 10.2), and lymphoma (adjusted OR: 2.43; 95% CI, 1.78 to 3.24) were at higher breakthrough risk. These hematologic cancers also had greater breakthrough risks than solid cancers (Appendix Fig A1C and Data Supplement).

Given that hematologic cancers were susceptible to breakthrough infection, we assessed the vaccine protection for each hematologic cancer type (Appendix Fig A1D and Data Supplement). Full vaccination protected patients with multiple myeloma (adjusted OR: 0.19; 95% CI, 0.1 to 0.35), lymphoma (adjusted OR: 0.19; 95% CI, 0.1 to 0.38), and lymphoid leukemia (adjusted OR: 0.11; 95% CI, 0.05 to 0.24) better from COVID-19 breakthrough. Moderna's vaccine showed stronger protection for patients with multiple myeloma (adjusted OR: 0.35; 95% CI, 0.15 to 0.72).

### Vaccination Effectiveness in Reducing Severe COVID-19 Outcomes in Patients With Cancer

**FIG A2.**
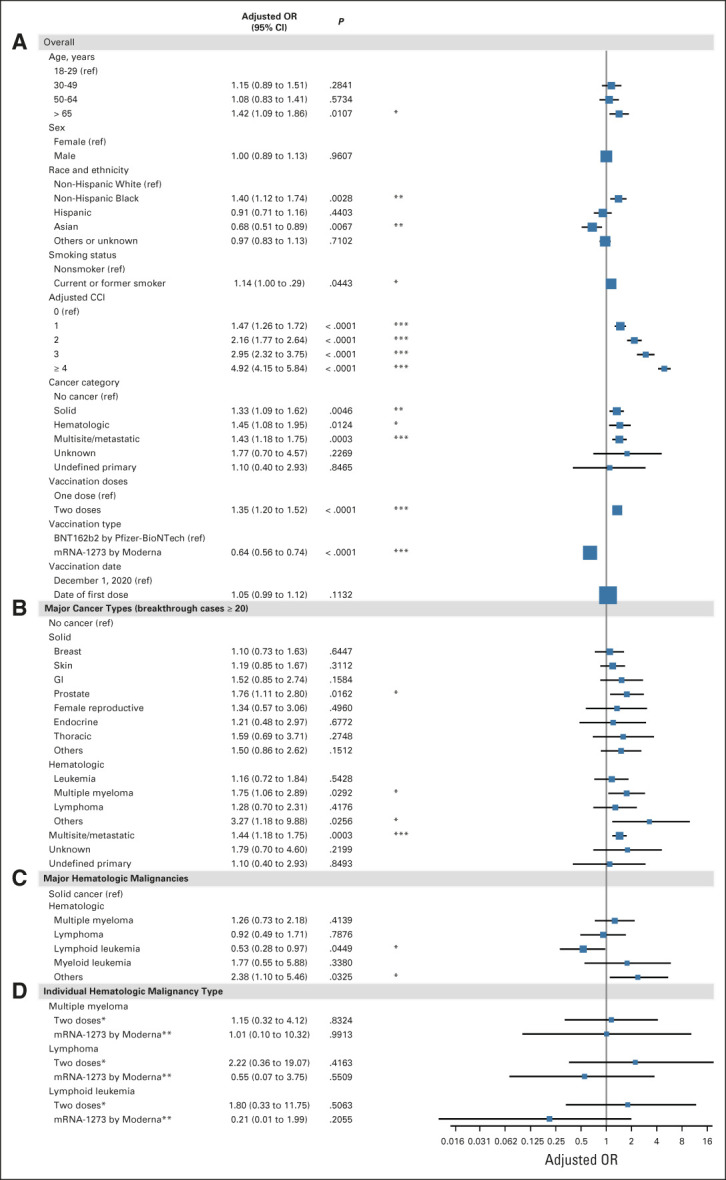
The mRNA vaccination effectiveness in reducing the risk of severe COVID-19 outcomes in patients with cancer. The forest plot of logistic linear regression analyses is shown for (A) all features in the overall breakthrough cases; (B) major cancer types in the overall breakthrough cases; (C) major hematologic malignancies (breakthrough cases ≥ 20) in breakthrough patients with cancers; and (D) fully vaccinated versus partially vaccinated cases and mRNA-1273 versus BNT162b2 vaccines in four individual patient groups with multiple myeloma, lymphoma, lymphoid leukemia, and myeloid leukemia. Age, sex, race and ethnicity, smoking status, adjusted CCI, vaccination doses, vaccination types, and vaccination date were included in all logistic regression models. Individual logistic regression analyses were performed for each hematologic malignancy type, and the results are summarized in (D). *P < .05, **P < .01, ***P < .001. CCI, Charlson Comorbidity Index; OR, odds ratio; ref, reference.

Age over 65 years, non-Hispanic Black patients, current or former smokers, and patients with more comorbidities (higher adjusted CCI) were at greater risk of severe COVID-19 outcomes (Appendix Fig A2A, online only). Meanwhile, compared with noncancer cases, solid cancers (adjusted OR: 1.33; 95% CI, 1.09 to 1.62) and hematologic cancers (adjusted OR: 1.45; 95% CI, 1.08 to 1.95) had higher risks of severe COVID-19 outcomes. Fully vaccinated cases were related to a higher risk of severe outcomes than partially vaccinated cases (adjusted OR: 1.35; 95% CI, 1.2 to 1.52), whereas patients with Moderna's vaccine (adjusted OR: 0.64; 95% CI, 0.56 to 0.74) had lower risks than Pfizer's.

Individual multivariable analyses regarding specific cancer types suggested that, compared with noncancer cases (Appendix Fig A2B, Data Supplement), patients with multiple myeloma were associated with increased risks of severe COVID-19 outcomes (adjusted OR: 1.75; 95% CI, 1.06 to 2.89). When compared with solid cancers (Appendix Fig A2C and Data Supplement), patients with lymphoid leukemia (adjusted OR: 0.53; 95% CI, 0.28 to 0.97) were at decreased risk.

Among individual hematologic cancers (Appendix Fig A2D and Data Supplement), although not significant, two doses of vaccination showed less protection for patients with multiple myeloma (adjusted OR: 1.15; 95% CI, 0.32 to 4.12), lymphoma (adjusted OR: 2.22; 95% CI, 0.36 to 19.07), and lymphoid leukemia (adjusted OR: 1.80; 95% CI, 0.33 to 11.75). Moderna's vaccine demonstrated stronger protection for patients with lymphoma (adjusted OR: 0.55; 95% CI, 0.07 to 3.75) and lymphoid leukemia (adjusted OR: 0.21; 95% CI, 0.01 to 1.99).

### Effects of Cancer Treatments on Vaccine Effectiveness

**FIG 2.**
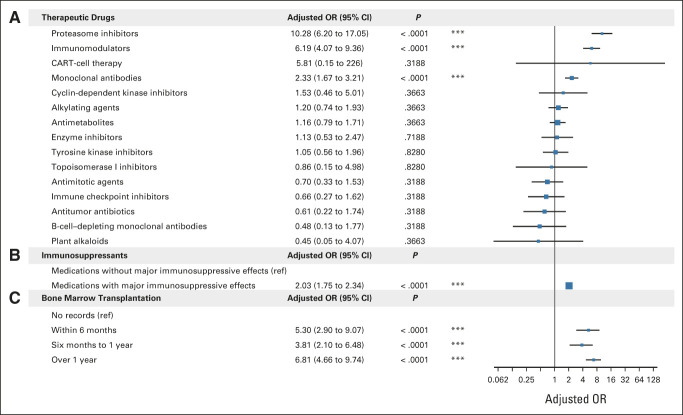
The effects of recent cancer treatments on breakthrough infection. The forest plot of logistic linear regression analyses is shown for (A) recent use (6 months before vaccination) of 15 NCI-derived drug categories in breakthrough infection cases, (B) recent use (6 months before vaccination) of drugs with *v* without major immunosuppressive effects in breakthrough infection cases, and (C) bone marrow transplantation in breakthrough infection cases. Age, sex, race and ethnicity, smoking status, vaccination doses, vaccination types, and vaccination date were included in all logistic regression models. Results from 14 separate logistic regression analyses, each tested a single medication category with its own reference group, are summarized in (A). The *P* values and 95% CIs shown in (A) were adjusted using FDR for multiple testing. *P < .05, **P < .01, ***P < .001. CAR, chimeric antigen receptor; FDR, false discovery rate; OR, odds ratio; ref, reference.

Cancer therapeutics and bone marrow transplantation procedures were systematically investigated using treatment-specific logistic linear regressions (Fig 2 and Data Supplement). The use of proteasome inhibitors and immunomodulators was significantly associated with higher breakthrough infection risks (adjusted ORs of 10.28 and 6.19, respectively). Medications with major immunosuppressive effects were associated with higher breakthrough risks (adjusted OR = 2.03). The history of bone marrow transplantations was associated with less vaccine effectiveness (range from adjusted ORs of 3.81-6.81).

### Further Analysis

We also examined the breakthrough risk, the severe outcome risk, and cancer treatment effects in patients who received two doses of vaccines (Data Supplement). The impact of recent cancer treatment on the breakthrough infection risk was explored (Data Supplement). The potential confounding effects because of the geographic variance were also checked (Data Supplement). The breakthrough infection risk of female- and male-specific cancers is explored in the Data Supplement. Stratified analysis was performed to reveal the treatment effects in hematologic malignancies (Data Supplement). The geographic distributions of cohort characteristics used in this work are presented in the Data Supplement. Goodness of fit and explained variations are provided in the Data Supplement. The above results are explained and discussed in the Data Supplement, and the variable selection results are provided in the Data Supplement. Main conclusions remained the same in these analyses.

## DISCUSSION

We reported the effectiveness of COVID-19 vaccines in patients with cancer using the largest national COVID-19 electronic medical record resource. Our results showed that patients with hematologic malignancies, especially multiple myeloma, were at a higher risk for breakthrough infection compared with noncancer patients or patients with solid tumor. Our findings aligned with recent serologic evidence in clinical studies and trials, which showed that the postvaccination antibody titers after vaccination were lower in patients^9,28-30^ with cancer including multiple myeloma,^31,32^ compared with those without cancers. The evidence suggested that the weaker serologic responses of patients with cancer to COVID-19 vaccines led to a higher risk of breakthrough infection.

Reducing health disparity is crucial for preventing COVID-19 breakthrough infections. Our study showed that non-Hispanic African Americans, including those with cancers, had significantly lower risks of breakthrough infections. Similar observations have been previously reported in smaller cohorts.^33-35^ However, the vaccination rate of African Americans was lower than other races,^36^ largely because of hesitancy.^37^ Our results underscored the urgency and importance of addressing COVID-19 vaccination hesitancy and increasing vaccination coverage in African Americans.

Our analysis suggested that vaccination protected patients with cancer, including those with hematologic malignancies, from breakthrough infections. Recently, the CDC approved the booster dose for immunocompromised individuals, including those with hematologic malignancies,^38^ and the corresponding data about the efficacy of booster doses are under collection at N3C. Whether our discoveries can be generalized to patients receiving booster shots is unknown. We will report the effectiveness of booster shots in follow-up studies once the data are available.

Despite lower breakthrough infection risk, fully vaccinated individuals had higher risk of developing severe outcomes compared with partially vaccinated individuals. These findings differ from those observed from other vaccine effectiveness studies including the study by Dagan et al,^39^ which reported a lower rate of COVID-19–related severe outcomes, largely because of the different definition of the at-risk population. Our study focused on the severe outcomes among breakthrough infection cases, whereas Dagan et al^39^ focused on the severe outcomes among all vaccinated population, including those who were not infected. Moreover, the N3C-vaccinated cohort had a larger proportion of patients with cancer compared with the cohort of Dagan et al^39^ (10% *v* 2%), which may explain the higher rates in our study. Furthermore, compared with partially vaccinated individuals in our N3C cohort, fully vaccinated individuals were older (median age 63 *v* 52 years), had higher comorbidities (22% *v* 12% of adjusted CCI ≥4), and were more likely to be smokers (40% *v* 20%), which also contributed to a higher rate of severe outcomes.

To our knowledge, our study is the first to provide RWE on the differential effects of mRNA vaccine types in specific cancer types. In both the general population and those with cancers, the mRNA-1273 by Moderna demonstrates better protection against breakthrough risk than Pfizer-BioNTech's BNT162b2 vaccine, consistent with the antibody-level difference in clinical trials.^18^ Moderna's vaccine is particularly effective for those with multiple myeloma (adjusted OR = 0.35) than the general population (adjusted OR = 0.66). However, such a difference is less obvious in other hematologic malignancies.

It is important to allow sufficient time for N3C's contributing sites to report vaccination status, breakthrough infections, and associated clinical outcomes. A 3-month reporting window was used in this study, using the data released on August 27, 2021, to study the COVID-19 breakthrough infections occurred before May 31, 2021. Our study mainly covered the COVID-19 Alpha (B.1.1.7) variant, as by the end of May 2021, delta variants had just started to emerge in the United States, with a proportion of 7.3% among new cases.^40^ Therefore, our study established the baseline for investigating infections of new COVID-19 variants such as the delta or the omicron variant.

The causes of the observed associations between cancer treatments and breakthrough infection risks are complicated. For example, proteasome inhibitors,^11,41^ immunomodulators, and bone marrow transplantations^42,43^ are commonly used to treat hematologic malignancies such as multiple myeloma,^42^ which by itself is strongly associated with breakthrough infections. Stratified analyses were performed for specific drug categories in certain hematologic malignancy types, the sample size of which was allowed (Data Supplement).

Our study was carefully designed to address known limitations in real-world data-based observational studies, including the reporting bias, the longitudinal continuum of cancer treatments, the design of the study, and selection of the statistic models. Our strategies are discussed in the Data Supplement. Comprehensive analysis on explained variations and goodness of fit suggested that, in general, our models explained the variation in the data well.

In conclusion, our work provided RWE suggesting that patients with cancer, especially those with hematologic malignancies such as multiple myeloma and lymphoma, were at higher risk of breakthrough infections after mRNA vaccinations and were more likely to develop severe outcomes. The COVID-19 mRNA vaccines still significantly reduced the breakthrough risk for patients with cancer. This work also provided a baseline for further investigating the efficacy of booster shots and the breakthroughs and outcomes of SARS-CoV-2 omicron variants infections.

## Data Availability

Qualified researchers may request Ono Pharmaceutical to disclose individual patient-level data from clinical studies through the following website: https://www.clinicalstudydatarequest.com/. For more information on Ono Pharmaceutical's Policy for the Disclosure of Clinical Study Data, see the following website: https://www.ono.co.jp/eng/rd/policy.html.
